# Dr. W. J. C. Keats on Practical Points in Infirmary Work

**Published:** 1913-11-22

**Authors:** W. J. C. Keats

**Affiliations:** Medical Superintendent, Camberwell Infirmary


### PRACTICAL POINTS IN INFIRMARY WORK.

**Dr. W. J. C. KEATS, Medical Superintendent, Camberwell Infirmary, gives his Views.**

After his discussion of "Problems of Discipline in Infirmary Work," published last week, Dr. Keats gave some interesting practical details concerning his institution.

"In the wards," he said, "you will note the patients' lockers, of which we are justly proud. Being original in design, original enough to be copied by other institutions, I need not say that they are home made. In the shape of a chair, the 'seat' part forms a box or locker, of which the top side forms the lid. The part corresponding to the back of the chair is several inches thick, surmounted by a porcelain slab and divided into two shelves by a centrepiece. The side of each shelf is open, without door of any kind. Sputum cups and other such appliances can be kept there. The back of the chair, on the side against which one's body would rest, possesses a flap which can be made into a table by the two segmental supports being moved outwards, and the height of the table is arranged so that when in use there is space enough for the whole thickness of bed and bed-clothes to pass beneath it and the top of the lower locker. Meals can thus be served on a table over the patient's knees when he is lying in bed. The woodwork is oak, and the design as simple as it is satisfactory."

### Timing the Dryers.

In the laundry Dr. Keats pointed out the rotatory dryers, which, like basins, dry the clothes by squeezing them against their sides, the pressure being gained through the centrifugal force generated by their whirling movement. Each dryer is provided with a clock, or rather, with the face of a clock, which is set when the machine, already filled with linen, is set in motion, the time recorded being the minute at which the clothes will be dry. In practice it has been found that after a quarter of an hour no more drying can be effected. If the clothes are put in at, say, 11, the attendant will set the clock at 11.15.

### A Save on the Coal Bill.

In the boiler-house Dr. Keats explained how "£1,500 a year has been saved on the infirmary's coal bill by using a hot-well in conjunction with a 'Green's Economiser.' Originally the condensed water was intended to be passed off into the sewer, but on the suggestion of Mr. Merrill, the engineer, the Guardians were persuaded at an expenditure of £50 to install these improvements.

### The Management of Gas-ovens.

In the kitchen Dr. Keats called attention to two interesting points concerning the gas-ovens. "Notice, first," he said, "the inside of one of these ovens. Look at the white tiles with which they are lined, and their cleanliness and fresh appearance. Yet they have been in use ten years. The reason why the tiles are not incrusted with brown stains and the accretions of years is that every time before each oven is used the tiles are scrubbed with limewash. One reason (he added) why there is a general prejudice against gas-ovens and gas-cooking generally, is that not sufficient care is taken to provide an exit for the burnt gas-fumes. A twisted, tortuous vent-pipe is a very cumbrous way of preventing down draught; it spoils the food and the oven. Such pipes we had here till I had them changed to the straight vertical chimney that you see here.

### The Secret of Floors.

"But before we leave the kitchen, look at the floor. It is the largest single expanse of uncracked granolithic that I know of anywhere. It has been down for eleven years, and there is no crack in it. Its dimensions are 25 feet by 30 feet. The secret of floors, however, is not the substance, but the laying. This is laid direct on concrete. The same material is in the theatre. There it has cracked because the iron girders on which ours, like most upper floors, is supported, expand and contract with the heat, and the floor cracks in consequence. The ward floors are of teak, and the corridors of a red granolithic, which has generally worn well, but it is very largely a matter of laying. Incidentally, one of the best ward floors I know is that in the wards of the Brook Fever Hospital.

### A Fine Mortuary Unit.

"I must, lastly, show you the mortuary unit, which has been admirably designed by Mr. Hall. First of all, it is at the back of the infirmary, with no houses opposite its street entrance, in an unfrequented street, though the Town Hall is only a few yards away. Secondly, a hearse-yard has been provided under cover, like a garage yard, where the hearse may enter to receive the coffin decently and in order unobserved from without. That was a good idea of Mr. Hall's. The mourners' rooms open out on one side, and on the other are the post-mortem rooms—we have about 240 'p.-m.s' a year—and the mortuary proper. Here, in place of the bins or slabs, are sliding compartments on runners with a door and label on each, and of a size to receive the body in a coffin. Everything here is designed so far as possible not to offend the sensibilities of any relatives and friends who may have cause to attend. Its extreme simplicity and effectiveness for its purpose from every point of view warrants, I think, this detailed description."

